# Molecular Aspects of Cellular Dysfunction in Alzheimer’s Disease: The Need for a Holistic View of the Early Pathogenesis

**DOI:** 10.3390/biom11121807

**Published:** 2021-12-01

**Authors:** Sara Merlo, Simona Federica Spampinato, Dmitry Lim

**Affiliations:** 1 Department of Biomedical and Biotechnological Sciences, Section of Pharmacology, University of Catania, 95123 Catania, Italy; simona_spampinato@hotmail.com; 2 Department of Pharmaceutical Sciences, Università del Piemonte Orientale, 28100 Novara, Italy

Alzheimer’s disease (AD) is the most common cause of dementia in the elderly, a socio-economic burden destined to worsen with increased population aging [1]. Although the neuronal mechanisms involved in the onset and progression of the disease have been largely deciphered, they are not sufficient to fully explain AD pathogenesis. Thus, for two decades, no new disease-modifying drug has reached approval until this year, when the FDA granted accelerated approval of the anti-beta amyloid antibody aducanumab [2]. While awaiting post-marketing evidence to fully confirm the clinical efficacy of this drug, the challenge of defeating AD remains open. The complex nature of AD pathogenesis and its slow development, with decades elapsing between the biochemical onset and the clinical stage, underlie the high rate of failed drug trials. The large number of gradual changes, which involve, although perhaps in different ways, all cellular types in the central nervous system (CNS), accumulate over this period, leading to a progressive disruption of the very delicate balance that grants CNS homeostasis [3,4,5]. By the time that symptoms appear, CNS damage is too extensive to allow for an efficient recovery. For this reason, new strategies have been more focused on the identification of reliable biomarkers for earlier diagnosis, which is critical for preventive approaches. Reaching this goal will require further understanding of the molecular complexity in the early time-window of AD pathogenesis, including cell-specific molecular dysfunctions that may, in turn, impact the cross-talk between different CNS cell types.

Our Special Issue comprises two original research articles and three reviews that, looking at different aspects, all contribute to improving and highlighting progress in the aforementioned topical aspects in the field of AD research. Comorbid conditions are discussed for both their potential as early diagnostic tools and as additional targets for AD-purposed drugs. Moreover, the targeting of transcriptional events, which occur at the neuronal level and involve hallmark proteins in AD pathogenesis, is proposed as a new tool for intervention. Finally, attention is focused on the emerging dichotomy of glial cells as mediators of either protective or neuroinflammatory effects during the time course of disease.

AD is associated with comorbid conditions of a different nature, with important implications from both diagnostic and therapeutic perspectives. As reviewed by Chunyan Liao et al. [6], AD patients show a high incidence of retinal aberrations, including electroretinographic changes, retinal thinning and vascular alterations. Notably, retinal degeneration appears to occur in early-stage AD. Consistently, both animal and human studies suggest an early appearance of both β-amyloid (Aβ) and hyperphosphorylated tau protein deposits in selective retinal layers. These are associated with damage and apoptosis of retinal ganglion cells and in the optical nerve. Based on these observations, monitoring retinal changes could represent an innovative means for the preclinical detection of subjects at higher risk for AD. However, this possibility is hindered by the presence of other ophthalmological disorders, such as age-related macular degeneration and glaucoma, which are unrelated to AD but share some molecular features. From here, the necessity of attentively distinguishing these pathologies from AD before adequate retinal biomarkers can be established.

Depression is often associated with AD and it contributes to worse clinical outcomes. Thus, the possibility of exploiting the drugs used for AD treatment to also ameliorate concomitant depressive states is an intriguing one. In the work by Morgese et al. [7], the natural food supplement 3-O-acetyl-11-keto-boswellic acid (AKBA), which was previously studied for its beneficial effects in AD models, was tested for its anti-depressive potential. The authors used an AD mouse model obtained by Aβ cerebral injection and verified the induction of a depressive-like state by behavioral and biochemical analyses. They analyzed the effects of AKBA treatment on these animals. The results showed that AKBA was able to prevent Aβ-induced depression, with a mechanism linked to inhibition of nuclear factor-kB (NF-kB) and a reduction in excitotoxicity and astrocyte activation. These results provide further evidence on the potential of food supplements as adjuvants in the treatment of AD and related depression.

Looking at the less-known functions of key protein determinants in AD pathogenesis may offer new clues for intervention, as discussed by D’Andrea et al. [8] in their review. Emerging evidence has shown that the APP-intracellular domain (AICD), the amyloid-β protein (Aβ), tau and ApoE can all localize at the nucleus, where they modulate the transcription of key molecules involved in AD pathogenesis, as well as each other, in feedback regulatory mechanisms. AICD regulates Aβ-degrading enzymes (neprilysin), pro-apoptotic genes (GSK-3β/β-catenin, p53, FOXO-3a) and mitochondrial genes. Aβ can enter the nucleus with yet unidentified mechanisms and directly bind DNA at a consensus sequence found in the APP, BACE and ApoE genes, while the nuclear functions of tau depend upon its dephosphorylation and are implicated in protective responses against nucleic acid-damaging stressors, as well as the regulation of nuclear calcium levels and CREB activation. Moreover, tau is implicated in regulating heterochromatin structure at specific regions, so tau dysfunction at the nucleus can result in aberrant gene expression. Finally, nuclear ApoEε4 can modulate the transcription of AD-related genes in both glial and neuronal cells, including the induction of APP transcription and repression of protective SIRT1- and BDNF-encoding genes. Overall, transcriptional defects emerge as novel and therapeutic targets in AD that have perhaps been overlooked to date.

The initial neuronocentric view of AD etiology was fully replaced by the evidence that the glial counterpart is equally involved in disease pathogenesis. In this context, the original work by Moelgg et al. [9] adds to the evidence of an early beneficial role for microglia, exploiting a sophisticated in vitro model consisting of organotypic brain slices, combined with the use of Aβ-loaded collagen hydrogels to obtain a controlled release of the peptide. Using this system, the authors showed that toxic Aβ42 could gradually spread between different brain areas, an effect enhanced in cultures from AD mouse models. Both astrocytes and microglia appeared to be highly activated in response to Aβ spreading, but only microglia appeared to operate an efficient clearance of the peptide, which is likely responsible for the prevention of plaque formation and cholinergic neuronal death.

To complete the picture of glial involvement in AD, Valenza et al. [10] provided a review of the most recent evidence regarding the role of astrocytes, looking at both the advantages and drawbacks of targeting these cells as a therapeutic option in AD. In particular, the authors underline the difficulty of choosing the right time for an intervention on astroglia, given their complex and highly context- and time-dependent phenotypic responses. Aging is associated with cellular senescence, which, in astrocytes, leads to the loss of neurotrophic features in favor of pro-inflammatory ones. As astrocytes maintain homeostasis at many different levels, their dysfunction affects a high number of cellular events, including neurotransmitter re-uptake, metabolic support to neurons and the release of neurotrophic cues. Many drugs that aim to rescue each of these impaired functions are under study and may offer valid therapeutic tools. Moreover, astrocytes are central mediators of neuroinflammation and oxidative stress, involving a wide variety of signaling pathways that, with the right timing, are all potential targets for intervention. Finally, the authors conclude with a dedicated focus on palmitoylethanolamide, a natural compound with a high safety profile, which was proposed as a modulator of astrocytic function, with anti-inflammatory and neuroprotective functions.

In conclusion, evidence from previous decades suggests that the preclinical and prodromal phases of AD pathogenesis represent the only window of opportunity for the early diagnosis and development of preventive or disease-modifying therapies. This approach requires a look out of the shell of the neuron-centric paradigm and the canonical Aβ hypothesis. Moreover, due to the complexity of the pathogenesis, as well as its multi-cell, multi-region and even multi-organ nature, in concomitance with co-morbid conditions and susceptibility to environmental factors, a holistic approach to the comprehensive investigation of the preclinical/prodromal AD phases is required. The contributions to this Special Issue are exemplary in illustrating the spectrum of phenomena the scientific community must address to find a cure for AD (Figure 1).

## Figures and Tables

**Figure 1:**
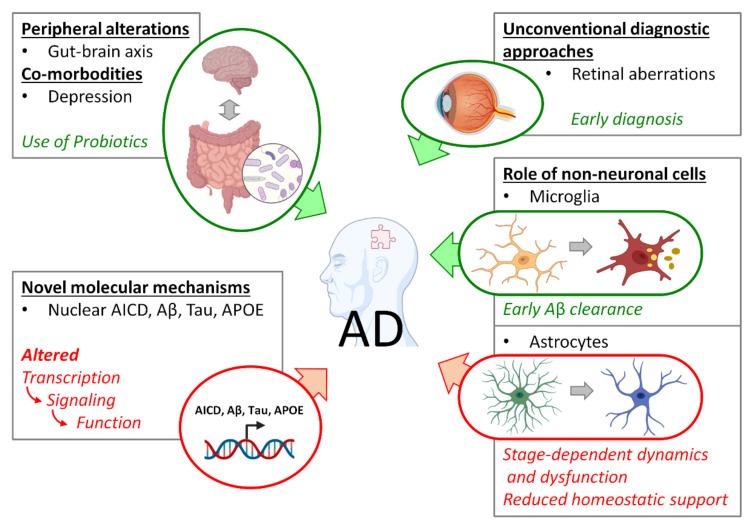
Spectrum of early AD-related phenomena addressed in this Special Issue. Green forms and arrows indicate positive interactions; red forms and arrows indicate negative interactions.
